# Morphometric Analysis of Lateral Masses of Axis Vertebrae in North Indians

**DOI:** 10.1155/2014/425868

**Published:** 2014-08-24

**Authors:** Monika Lalit, Sanjay Piplani, J. S. Kullar, Anupama Mahajan

**Affiliations:** ^1^Department of Anatomy, Sri Guru Ram Das Institute of Medical Science and Research, Vallah, Amritasr 143001, Punjab, India; ^2^Department of Pathology, Sri Guru Ram Das Institute of Medical Science and Research, Vallah, Amritasr 143001, Punjab, India; ^3^Department of Anatomy, Government Medical College, Amritsar, Punjab, India

## Abstract

*Background and Objective.* The lateral masses of axis have good cancellous bone quality beneath the articular surface of facets that make this area a good site for the insertion of an internal fixation device.* Methods.* 60 dry axis vertebrae were obtained for anatomic evaluation focused on pedicle, superior and inferior articular facets, and foramen transversarium. Based upon linear and angular parameters the mean, range, and standard deviation were calculated. *Results.* The mean length, width, and height of the pedicle were 21.61 ± 2.37 mm, 8.82 ± 2.43 mm, and 5.63 ± 2.06 mm. The mean pedicle superior angle and median angle were 23.3 and 32.2 degrees. The mean superior articular facet length, width, and external and internal height were 16.34 ± 1.56 mm, 14.35 ± 1.75 mm, 8.98 ± 1.36 mm, and 4.23 ± 0.81 mm. Depth of vertebral artery was 4.72 ± 0.83 mm. Mean inferior articular facet length and width were 11.13 ± 1.43 mm and 7.89 ± 1.30 mm. The mean foramen transversarium length and width were 5.11 ± 0.91 mm and 5.06 ± 1.23 mm.* Conclusions.* The study may provide information for the surgeons to determine the safe site of entry and trajectory for the screw implantation and also to avoid injuries to vital structures while operating around axis.

## 1. Introduction

Axis, the second cervical vertebra, forms a pivot on which the atlas rotates carrying the head to allow greater range of motion at the atlantoaxial joints [1, 2]. The lateral masses of axis have good cancellous bone quality beneath the articular surface of facets that makes this area a good site for insertion of an internal fixation device [3].

The elements of pedicle and pedicle axis are critical to the structural anatomy of axis vertebra which are important to normal function and also for cases of pathology or fractures when surgical intervention is required [4, 5]. The superior articular facets (SAF) of axis differ from other vertebral facets which make this region more prone to vertebral artery injury during screw fixation [6]. In axis vertebrae the SAF lies in proximity to the body and medial aspect of the axis of the pedicle whereas SAF of other vertebrae are lying in proximity to the junction of pedicle and lamina and also the vertebral artery foramen is lying partially or completely in the undersurface of axis while in other vertebrae the vertebral artery foramen is located entirely in relation to foramen transversarium [7, 8]. This unusual location of vertebral artery foramen makes the vertebral artery more prone to injury during screw fixation [9].

The present study may also assist with placement of screws into pedicle or lateral mass. For this reason, the posterior point of projection of the pedicle axis has been defined in terms of reference points visible in a postsurgical approach to the cervical spine and the two angles represent the safe bounds for transpedicular screw fixation/placement [4, 10]. Thus if there is any variation in the lateral mass of axis or associated anomalous vertebral artery, it may lead to thinning of lateral mass and pedicle would prevent adequate fixation of transpedicular screw and vertebral artery will also be at risk [2].

Therefore, the present study was designed to know the dimensions of lateral masses of axis that are important to analyze their relationship with the vertebral artery and also to determine the ideal drill angle for accurate placement of a screw in the area resulting from fracture or partial sublaxation [4]. Thus it would be essential for the clinicians and surgeons to have a proper orientation of the anatomy, dimensions, and special features of this unique vertebra.

## 2. Material and Methods

**Figure 1:**
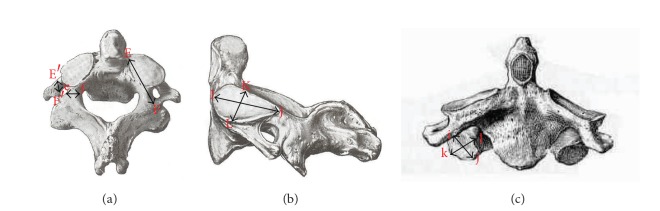
(a) Superior view of axis showing pedicle length (PL = EF), pedicle height (PH = E′F′) and pedicle width (PW = ef). (b) Lateral view of axis showing superior articular facet length (SAFL = IJ) and width (SAFW = KL). (c) Anterior view of axis showing inferior articular facet length (IAFL = ij) and width (IAFW = kl).

**Figure 2:**
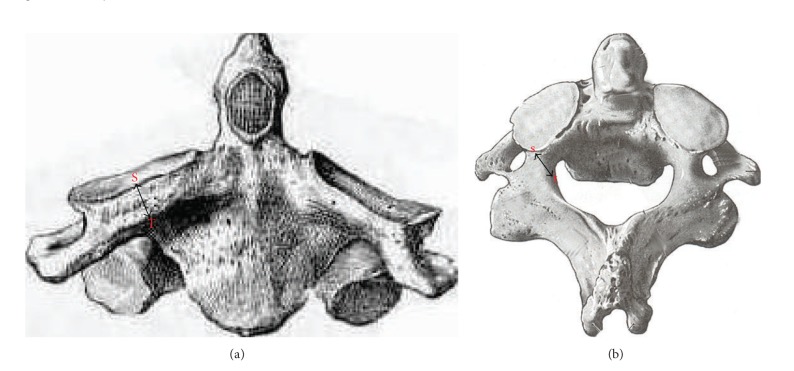
(a) Anterior view of axis showing superior articular facet external height (ST = SAFH_E_). (b) Superior view of axis showing superior articular facet internal height (SAFH_I_ = st).

**Figure 3:**
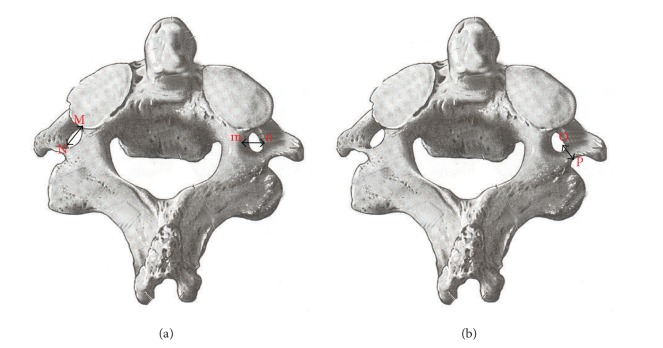
(a) Superior view of axis showing foramen transversarium length (FTL = MN) and width (FTW = mn). (b) Superior view of axis showing foramen transversarium depth (FTD = OP).

**Figure 4:**
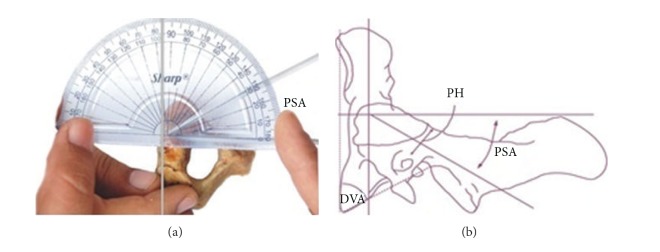
Showing superior angle of pedicle axis projection marked as PSA.

**Figure 5:**
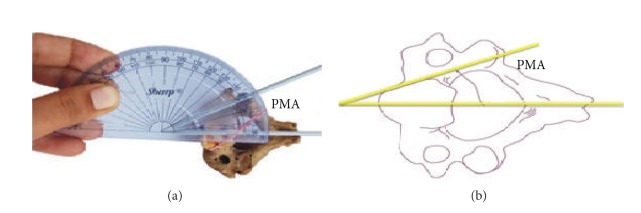
Showing median angle of pedicle axis marked as PMA.

The study was conducted on 60 dry axis vertebrae obtained in the Department of Anatomy, Government Medical College, Amritsar. The measured parameters included dimensions regarding lateral masses of axis vertebrae focused on pedicle, superior articular facet, inferior articular facet, foramen transversarium and pedicle angles. Fifteen parameters were measured and all the measurements were made using a vernier caliper accurate to 0.1 mm. The angles were measured by using an iron wire, scale, and a protractor. Based upon linear and angular parameters the mean, range, and standard deviation were calculated. The statistical analysis of the measurements of right and left sides was also done (Table 1 and Figures 1, 2, 3, 4, and 5).

To measure the angles first point of pedicle axis projection would be defined which was determined by drawing two lines parallel to the axis, one on superior surface of the pedicle and the other on medial side of the pedicle. These two lines intersected on the posterior aspect of the lateral mass.

## 3. Results

The measurements of all parts of lateral mass of axis vertebrae including the angles showed uniformity and there was no significant statistical difference observed in the mean dimensions of the parameters measured. The pediclelength, width, and height were 21.61 ± 2.37 mm, 8.82 ± 2.43 mm, and 5.63 ± 2.06 mm, respectively. Length and width of superior articular facet were 16.34 ± 1.56 mm and 14.35 ± 1.75 mm. External and internal height were 8.98 ± 1.36 mm and 4.23 ± 0.81 mm. Thus 4.72 ± 0.83 mm is depth of vertebral artery calculated as the difference between external and internal height. Length and width of inferior articular facet were 11.13 ± 1.43 mm and 7.89 ± 1.30 mm. The foramen transversarium length, width, and depth were found to be 5.11 ± 0.91 mm, 5.06 ± 1.23 mm, and 4.09 ± 0.74 mm, respectively. Pedicle superior angle was 23.3 degrees and pedicle median angle was 32.2 degrees (Table 2).

## 4. Discussion

Detailed knowledge of pedicle would be essential if transpedicular screw placement is desired to stabilize a fracture line through *C*_2_ pedicle [4, 14, 15]. Pedicle angles represent the safe bounds for transpedicular screw fixation.

A glance at Table 3 elucidates that, in the present study, the parameters of pedicle length, pedicle width, and pedicle height of right and left sides were compared and no significant statistical difference was observed in the mean dimension of the parameters studied on the two sides of the vertebrae. None of the earlier authors have compared the parameters of both the sides in the available accessible literature and the difference of mean of pedicle length (PL) was (*P* = 0.872), width (PW) was (*P* = 0.895), and height (PH) was (*P* = 0.886), respectively, which was found to be statistically insignificant.

The values of superior articular facet external height (SAFH_E_) reported in the present study stood equivalent to Madawi et al. (1997) [2] and Gupta and Goel (2000) [9] and the values of right and left sides were also calculated and the difference between mean dimensions of external height of both the sides was observed to be statistically insignificant (*P* = 0.488) whereas the superior articular facet internal height (SAFH_I_) in the present study stand equivalent to Madawi et al. (1997) [2] and Gupta and Goel (2000) [9] and the difference between the mean dimensions of internal height of right and left sides was also found to be statistically insignificant (*P* = 0.620). It is also interpreted in Table 3 that the findings of depth of vertebral artery in the present study stands equivalent to Madawi et al. (1997) [2] and Gupta and Goel (2000) [9] and are statistically not significant (*P* = 0.233).

A glance at Table 3 also showed that in the present study the difference of mean of foramen transversarium length (FTL) was (*P* = 0.482), width (FTW) was (*P* = 0.662), and depth was (FTD) (*P* = 0.645), respectively, which was found to be statistically insignificant and also these parameters showed no major difference when compared with the workers reported in the literature. The pedicle superior angle (PSA) in the present study was slightly less as compared with work done by Madawi et al. (1997) [2] and more to Xu et al. (1995) [4]. No significant statistical difference was observed in the mean dimension of the angles studied on the two sides of the vertebrae (*P* = 0.378) whereas the pedicle median angle (PMA) was in accordance to the work done by Xu et al. (1995) [4] and Madawi et al. (1997) [2]. No significant statistical difference was observed in the mean dimension of the angles studied on the two sides of the vertebrae (*P* = 0.211).

A glance at Table 4, in the present study the length of superior articular facet (SAFL) in North Indian population showed very slight difference when compared with the study of Francis (1955) [13], Xu et al. (1995) [4], and Gupta and Goel (2000) [9]. The difference between the mean of length of right and left superior articular facet of axis was found to be statistically insignificant (*P* = 0.141) and width of SAF (SAFW) in North Indian population showed very slight difference when compared with the study of Francis (1955) [13], Xu et al. (1995) [4] and Gupta and Goel (2000) [9]. The difference between mean of width of right and left superior articular facet of axis was found to be statistically insignificant (*P* = 0.661).

Values of inferior articular facet length of axis (IAFL) in the present study stood equivalent whereas inferior articular facet width (IAFW) was found to be less when compared with work done by Francis (1955) [13] and Gupta and Goel (2000) [9]. But the comparison of right and left sides was not specified by them. No significant statistical difference was observed in the mean dimension of the IAFL (*P* = 0.274) and IAFW (*P* = 0.681) studied on the two sides of the vertebrae.

## 5. Conclusions

The study may provide information for the surgeons to determine the safe site of entry and trajectory for the screw implantation and also toavoid injuries to vital structures while operating around axis. Dimensions of axis vertebral foramen transversarium are important and act as a useful guide in the estimation of dilation of vertebral artery. The vertebral artery and the basilar artery contribute blood supply not only to the brain but to inner ear also and their compression may lead to irritation of sympathetic plexus, manifested not only by neurological symptoms but also by labyrinthine or hearing disturbances. Thus dimensions of axis vertebral foramen transversarium are important and act as a useful guide in the estimation of dilation of vertebral artery [12, 16, 17]. SAF of axis has a crucial relationship with vertebral artery that makes the vertebral artery more prone to injury. Asymmetry of articular processes in particular hypertrophy of articular processes might have caused torticollis with severe constriction of cervical mobility [18]. To determine accurate placement of a screw in the area of any deformity resulting from fracture or partial sublaxation, ideal drill angle for transpedicular screw placement is required. Therefore careful anatomic reduction is essential [4, 19].

## Figures and Tables

**Table 1:** The measured parameters.

S. number	Parameters of lateral mass of axis vertebrae (pedicle, SAF, IAF, FT, and angles)	
1	Pedicle length (PL)	Length was measured from anterior most point of the pedicle axis to the posterior point of pedicle axis projection EF = PL (Figure 1(a)).
2	Pedicle width (PW)	It was taken from internal surface of pedicle to its external surface at the level of transverse foramen ef = PW (Figure 1(a)).
3	Pedicle height (PH)	It was measured from its superior surface to inferior surface with in the foramen transversarium E′F′ = PH (Figure 1(a)).
4-5	Superior articular facet length (SAFL) and width (SAFW)	It is the maximum anteroposterior and transverse diameter of articular surface of superior facet. (IJ = SAFL) (KL = SAFW) (Figure 1(b)).
6	External height (SAFH_E_)	It was taken from upper midpoint on the superior articular surface to the lower midpoint on the inferior surface. ST = SAFH_E _(Figure 2(a)).
7	Internal height (SAFH_I_)	It was taken from midpoint of articular surface to the nearest point on the inferior surface. st = SAFH_I_ (Figure 2(b)).
8	Depth of vertebral artery	It was taken as the difference between external and internal height.
9-10	Inferior articular facet length (IAFL) and width (IAFW)	It is the maximum anteroposterior and transverse diameter of articular surface of Inferior facet. (ij = IAFL) (kl = IAFW) (Figure 1(c)).
11–13	Foramen transversarium length (FTL), width (FTW), and depth (FTD)	It is the maximum anteroposterior, transverse, and superoinferior diameter. (MN = FTL) (mn = FTW) (OP = FTD) (Figures 3(a) and 3(b)).
14	Pedicle superior angle (PSA)	It was measured as the angle between the pedicle axis and a line drawn perpendicular to the body of the axis read as PSA (Figure 4).
15	Pedicle median angle (PMA)	It was measured as the angle between the pedicle axis and line passing through the midline of the vertebral body and the spinous process and read as PMA (Figure 5).

**Table 2:** Results of measured parameters of lateral mass of axis vertebra.

S. number	Parameters (mm)	Mean	SD	Range	*P* value
1	Pedicle length (PL)	21.61	2.37	16.00–25.15	0.872
2	Pedicle width (PW)	8.82	2.06	3.60–12.50	0.895
3	Pedicle height (PH)	5.63	2.43	2.20–13.95	0.886
4	Superior articular facet length (SAFL)	16.34	1.56	13.90–20.70	0.141
5	Superior articular facet width (SAFW)	14.35	1.75	11.40–17.70	0.661
6	Superior articular facet external height (SAFH_e_)	8.98	1.36	5.8–12.05	0.488
7	Superior articular facet internal height (SAFH_i_)	4.23	0.81	2.40–6.60	0.620
8	Depth of vertebral artery	4.72	0.83	2.40–6.05	0.233
9	Inferior articular facet length (IAFL)	11.13	1.43	8.80–14.50	0.274
10	Inferior articular facet width (IAFW)	7.89	1.30	6.70–10.20	0.681
11	Foramen transversarium length (FTL)	5.11	0.91	2.75–6.50	0.482
12	Foramen transversarium width (FTW)	5.06	1.23	3.25–8.00	0.662
13	Foramen transversarium depth (FTD)	4.09	0.74	2.70–5.30	0.645
14	Pedicle superior angle (PSA)	23.32	2.62	19.00–29.00	0.378
15	Pedicle median angle (PMA)	32.23	1.65	29.00–36.00	0.211

**Table 3:** Comparison of results reported in the present study and series reported in the literature.

Parameters	Lalit et al. (present study) 2014 North Indian 60	Xu et al. 1995 [4] Ohio 50	Madawi et al. 1997 [2] England 50	Gupta and Goel 2000 [9] Maharashtrian 50	Howington et al. 2001 [11] American 10	Taitz et al. 1978 [12] Israel 32
	Mean (mm) and S.D range (mm)	Mean (mm) and S.D range (mm)	Mean (mm) and S.D range (mm)	Mean (mm) and S.D range (mm)	Mean (mm) and S.D range (mm)	Mean (mm) and S.D range (mm)
PL	21.61 ± 2.37 16.00–25.15	Male (30) 25.6 ± 1.8 23–31 Female (20) 25.5 ± 1.4 23–29	28.7 ± 2.2 22–36	—	16.6	—

PW	8.82 ± 2.06 3.60–12.50	Male (30) 7.7 ± 1.4 5–10 Female (20) 9 ± 1.1 4–10	7.9 ± 1.5 4.7–12.4	7.9 4.8–14.5	9.1	—

PH	5.63 ± 2.43 2.20–13.95	Male (30) 8.6 ± 1.2 6–12 Female (20) 7.9 ± 1.4 6–10	7.8 ± 1.3 3.4–12.2	7.7 4.5–12	7.9	—

SAFH_E_	Rt. 8.87 ± 1.63 5.5–13.90 Lt. 9.08 ± 1.51 5.6–13.30 Mean 8.98 ± 1.36 5.8–12.05	—	8.6 ± 1.4 5.3–12.3	8.4 5.5–11.5	—	—

SAFH_i_	Rt. 4.27 ± 0.88 2.70–6.50 Lt. 4.21 ± 0.86 2.20–7.10 Mean 4.23 ± 0.81 2.40–6.60	—	4.4 ± 1.8 0.8–9.0	4.62 1.5–8.5	—	—

Depth of vertebral artery	Rt. 4.61 ± 1.09 2.60–7.40 Lt. 4.87 ± 0.95 2.20–6.60 Mean 4.72 ± 0.83 2.40–6.05	—	4.2 ± 1.6 1.0–9.4	4.36 2.0–8.2	—	—

FTL	Rt. 5.03 ± 1.07 3.00–7.00 Lt. 5.18 ± 1.09 1.50–7.00 Mean 5.11 ± 0.91 2.75–6.50	—	—	—	—	Rt. 5.85 ± 1.39 3.9–11.3 Lt. 5.76 ± 0.71 4.0–7.3

FTW	Rt. 5.12 ± 1.32 3.00–8.00 Lt. 5.00 ± 1.53 2.00–8.00 Mean 5.06 ± 1.23 3.25–8.00	—	5.6 ± 0.9 4.3–8.0	—	—	Rt. 4.77 ± 0.70 3.6–6.2 Lt. 4.99 ± 0.66 3.4–6.0

FTD	Rt. 4.13 ± 0.87 2.50–5.80 Lt. 4.05 ± 0.89 2.40–5.80 Mean 4.09 ± 0.74 2.70–5.30	—	5.7 ± 1.0 3.9–9.0	—	—	—

PSA	Rt. 23.40 ± 2.63 19.00–29.00 Lt. 23.23 ± 2.71 19.00–29.00 Mean 23.32 ± 2.62 19.00–29.00	Male (30) 20.4 ± 4.0 14–30 Female (20) 20.0 ± 3.8 13–25	24.0 ± 4.2 13–34	—	—	—

PMA	Rt. 32.10 ± 1.95 29.00–37.00 Lt. 32.37 ± 1.52 29.00–36.00 Mean 32.23 ± 1.65 29.00–36.00	Male (30) 33.3 ± 2.9 26–40 Female (20) 32.7 ± 3.4 28–41	32.4 ± 4.0 24–43	—	—	—

**Table 4:** Comparison of superior and inferior articular facets length and width of axis vertebrae.

Worker and year	Population	SAFL mean (mm) and S.D range (mm)	SAFW mean (mm) and S.D range (mm)	IAFL mean (mm) and S.D range (mm)	IAFW mean (mm) and S.D range (mm)
Francis 1955 [13]	White males 109	Rt. 18.8 ± 1.6 15–23 Lt. 18.7 ± 1.5 15–23	Rt. 16.7 ± 1.6 14–22 Lt. 18.3 ± 1.7 15–23	Rt. 11.0 ± 1.7 8–18 Lt. 11.3 ± 1.5 9–16	Rt. 11.9 ± 1.4 9–16 Lt. 12.1 ± 1.5 9–17
	White females 27	Rt. 18.0 ± 1.8 14–20 Lt. 17.4 ± 1.4 14–20	Rt. 15.1 ± 1.3 13–18 Lt. 16.7 ± 1.7 12–20	Rt. 11.6 ± 1.3 9–14 Lt. 11.1 ± 1.4 9–14	Rt. 10.7 ± 1.1 9–13 Lt. 11.4 ± 1.2 10–15
	Negro males 135	Rt. 19.0 ± 1.3 16–22 Lt. 18.9 ± 1.5 16–22	Rt. 17.7 ± 1.4 15–21 Lt. 17.9 ± 1.4 15–21	Rt. 12.0 ± 1.7 9–16 Lt. 11.4 ± 1.8 8–16	Rt. 11.3 ± 1.6 8–15 Lt. 11.8 ± 1.4 9–15
	Negro females 57	Rt. 17.6 ± 1.3 15–20 Lt. 17.7 ± 1.5 15–21	Rt. 15.7 ± 1.1 14–18 Lt. 16.1 ± 1.4 13–19	Rt. 11.2 ± 1.1 9–13 Lt. 11.0 ± 1.2 9–14	Rt. 10.7 ± 1.1 9–14 Lt. 11.1 ± 1.1 9–13

Xu et al. 1995 [4]	Ohio males 30	18.2 ± 1.5 16–23	17.6 ± 1.3 16–21	—	—
	Ohio females 20	17.1 ± 1.1 15–19	16.9 ± 1.3 14–19	—	—

Gupta and Goel 2000 [9]	Maharashtrian 50	16.5 13–20.5	15.9 12–19.5	10.59 7–15	9.58 6–15

Lalit et al. (Present Study) 2014	North Indians 60	Rt. 16.49 ± 1.61 13.90–20.50 Lt. 16.18 ± 1.70 13.50–20.90 Mean 6.34 ± 1.56 13.90–20.70	Rt. 14.39 ± 2.01 11.00–18.20 Lt. 14.31 ± 1.62 11.40–17.30 Mean 14.35 ± 1.75 11.40–17.70	Rt. 11.26 ± 1.46 8.70–14.00 Lt. 10.99 ± 1.68 7.80–15.00 Mean 11.13 ± 1.43 8.80–14.50	Rt. 7.83 ± 1.27 5.80–11.80 Lt. 7.94 ± 1.64 4.80–12.40 Mean 7.89 ± 1.30 6.70–10.20
